# Hospital Saturday Fund

**Published:** 1898-11-05

**Authors:** 


### The Institutional Workshop.

### HOSPITAL SATURDAY FUND.

Special interest attached to this year's annual meeting of the Hospital Saturday Fund, held on Saturday, October 29th, in the Egyptian Hall of the Mansion House, as being the first since the decision to discontinue the street collections which have hitherto formed so notable a feature in the Fund's operations. The meeting was a crowded one, despite a very wet day, and the assembled delegates seemed united in cheerful acquiescence in the momentous decision arrived at; the applause was hearty, and the resolutions were carried *nem. con.* In the absence of the Lord Mayor, detained at a more ceremonial function at Guildhall, Mr. Reginald B. D. Acland, the chairman of the Fund, presided, and opened the proceedings with his customary statement of accounts. He professed himself somewhat afraid of the reception he might get at the hands of the ladies on account of his having set his face against the street collections, but was quite prepared to give his reasons for his attitude. Beautiful and laudable as it was for them to go out and do this work, it was not expedient. The Fund was losing position and caste; the public was getting to dislike the idea extremely for reasons for which the directors were not responsible, and it had at length become clear to them that it was desirable to give up the street collections altogether. It was a bold step; they knew they were likely to lose money by it, and they had lost money, but it was a step in the right direction. They were confident that the loss would be made up in other ways, and already they had got into workshops where they could not get before, with the result that while last year £10,353 was collected in the workshops, this year already they had received £11,185—an increase of £832 during the first three quarters, while they hoped for a still larger increase during the remaining quarter, since it was the quarter in which they usually did best in that direction. On the other hand their expenses were £259 less, so that on the workshops they were £1,100 to the good. But last year the street collections produced £3,300, so that they had at present to face a loss of £2,000, of which they hoped to make up at least £1,000 before the end of the year. And if they could do that in the first year, when arrangements had not been completed so as to make it a fair test, and if they could gain at the same time the applause of those most interested and most entitled to speak, he thought they might consider the step fully justified.

The first resolution, "That this meeting, recognising the interest which the artisans of the metropolis are taking in the medical charities of London, confidently appeals to the working classes through the Hospital Saturday Fund for continued support, to obviate any loss to the Fund by the discontinuance of the annual Ladies' Street Collection," was moved by Sir Arthur Arnold, who said he regarded the Hospital Saturday Fund as by far the best and most important, and most useful of all the funds, because it taught the classes most benefited to rise to the beauty and dignity of self-help. It appealed to those to whom hospitals were an insurance against accident and disease, and asked them not to accept the boon without themselves contributing towards it. As regarded the street collections, their institution in London was due, in part, at least, to a suggestion of his own. He had seen for the first time in Dresden, in 1870, during the Franco-German war, ladies sitting in the streets soliciting aid for the sick and wounded, and when the Hospital Saturday Fund was started he suggested the adoption of the plan. But they knew what extended developments, what suspicious imitations had occurred. In presence of more imitations it was felt impossible that it could be continued, and he had felt constrained in his address to the London County Council in 1896 to call attention to the matter. If not checked it would degenerate into constant begging—an offence to passers-by, and a hindrance to traffic, which must eventually lead to prosecution by the police. Holding those opinions, he was glad that the committee had arrived at the decision they had.

Sir Henry Burdett said such a gathering as that, on a wet Saturday, showed that there were behind the Fund living men and women who intended to make it a success. Sir Arthur Arnold had referred to the question of the public authorities and street collections, and he quite agreed with him. It was due to the Fund that its courageous action should be followed up, and the matter no longer left in abeyance. If it required courage on the part of the committee to abandon the income derived from street collections, what must have been the committee's feeling as to the volume of public opinion on the subject! He asked the authorities to get rid of a dangerous nuisance. He wished the Lord Mayor had been there in person, so that the matter might have been pressed home on him directly, for he thought that the City of London should lead the way and institute a bye-law to put down street collections altogether; that was the least the Hospital Saturday Fund ought to expect from the authorities. After referring to an interview in the *Sunday Special* with Mr. Bunn, the secretary, which gave a very interesting account of the working of the Fund, and the reasons why it should be supported, Sir Henry went on to deal with the effect on that and other institutions of the Prince of Wales's Fund. This, he believed the evidence showed, could only be regarded as beneficial and encouraging. It had widened materially the area of interest in the hospitals. Returning to the resolution, he had a suggestion to make for getting increased support to the Fund. There were many workmen, among them some of the most thrifty, and numbers of independent artisans and men in a small way of business, who were not touched by the workshop collections. These, he felt, should have some record of any help they gave, and this could be managed if the district councils would have a stock of hospital stamps for the purpose, together with the bills setting out the method of using them, and so forth. Then givers of 1s. or other sums could have the amount recorded, the stamps could be kept in the albums issued for the purpose, and this could go on indefinitely. He would like to arrange to supply these stamps, free of cost, to the Fund, if they thought well of the suggestion. One other point he wished to deal with. The Council had had the courage to purchase most excellent premises, where the work could be properly carried out. That, of course, meant money; the cost had been £5,500, and of that £4,000 still remained to be raised. He should like to press this on the attention of the men who had more money than they knew what to do with to get them to indulge in the luxury of giving, especially in the case of wealthy employers of labour. He hoped they would look into the matter, and would take care that this £4,000 was paid in before the end of the year —it would be a most suitable Christmas-box for the Fund. As a small start, he was authorised by a friend to offer £25, to be increased to £50 if the whole amount were raised by December 31st. Concluding, Sir Henry said it was well known that he and some of those then with him on the platform had sometimes stood on opposite sides with regard to various questions connected with the Fund, but he was bound to say now that, as he understood its administration, there was no institution in better order or better deserving of large support. He had, therefore, great pleasure in seconding the resolution, which, after Dr. Gordon Brown had spoken in its favour, was carried unanimously.

Sir Savile Crossley moved the next resolution in favour of regular weekly contributions in workshops and places of business, and took the fact that he, the hon. secretary of the Prince of Wales's Fund, was entrusted with it as showing that the Council did not believe, as had been feared, that the two funds would clash. As a fact, they had succeeded in not clashing at all, and there was no reason why they should, or the Sunday Fund either, for they all had the same object.

The Hon. Sydney Holland supported the resolution in a humorous and forcible address. He was quite sure that working men in London did not support the hospitals to anything like the extent they should. Their idea seemed to be that the hospitals had gone on so long that they would continue to do so. They wanted the notion of the need for help brought home to them. As an instance of this, he mentioned that at the London and at the Poplar Hospitals, since they had made a small charge to cover the cost of bandages and medicines, the money-boxes that used to stand empty had had contributions dropped into them, and this went on and increased from year to year, while no man ever objected to the charge. He thought a great deal might be done to bring home the necessary sense of responsibility if employers would set the example and would consult their men as to instituting a penny weekly collection in the workshops. As to the complaints which were sometimes heard regarding the management of the hospitals, he said it was impossible always to avoid these. Why, people could not always manage their households and their wives without some complaint arising, and, speaking for the London Hospital, he would remind them that they had 11,000 in-patients, 167,000 out-patients, 300 nurses, and 350 students, and it was unavoidable that something should go wrong sometimes.

The Rev. G. W. Keesey and Mr. Hamilton Hoare also spoke, and the meeting concluded with votes of thanks to the Lord Mayor for the use of the hall, and to the chairman for presiding.

